# Ectoparasitic growth of *Magnaporthe* on barley triggers expression of the putative barley wax biosynthesis gene *CYP96B22* which is involved in penetration resistance

**DOI:** 10.1186/1471-2229-14-26

**Published:** 2014-01-14

**Authors:** Rhoda Delventhal, Christian Falter, Roxana Strugala, Nina Zellerhoff, Ulrich Schaffrath

**Affiliations:** 1 Department of Plant Physiology, RWTH Aachen University, 52056 Aachen, Germany; 2 current address: Biozentrum Klein Flottbek, Molecular Phytopathology and Genetics, Ohnhorststr. 18, 22609 Hamburg, Germany; 3 current address: Institute of Botany, University of Cologne, Zülpicher Str. 47b, 50674 Cologne, Germany

**Keywords:** Nonhost resistance, *Magnaporthe oryzae*, Head blast, Cytochrome P450, Wax, Cuticle, Penetration, BSMV-VIGS

## Abstract

**Background:**

Head blast caused by the fungal plant pathogen *Magnaporthe oryzae* is an upcoming threat for wheat and barley cultivation. We investigated the nonhost response of barley to an isolate of the *Magnaporthe* species complex which is pathogenic on *Pennisetum* spp. as a potential source for novel resistance traits.

**Results:**

Array experiments identified a barley gene encoding a putative cytochrome P450 monooxygenase whose transcripts accumulate to a higher concentration in the nonhost as compared to the host interaction. The gene clusters within the CYP96 clade of the P450 plant gene family and is designated as *CYP96B22.* Expression of *CYP96B22* was triggered during the ectoparasitic growth of the pathogen on the outside of the leaf. Usage of a fungicidal treatment and a *Magnaporthe* mutant confirmed that penetration was not necessary for this early activation of *CYP96B22*. Transcriptional silencing of *CYP96B22* using *Barley stripe mosaic virus* led to a decrease in penetration resistance of barley plants to *Magnaporthe* host and nonhost isolates. This phenotype seems to be specific for the barley-*Magnaporthe* interaction, since penetration of the adapted barley powdery mildew fungus was not altered in similarly treated plants.

**Conclusion:**

Taken together our results suggest a cross-talk between barley and *Magnaporthe* isolates across the plant surface. Since members of the plant CYP96 family are known to be involved in synthesis of epicuticular waxes, these substances or their derivatives might act as signal components. We propose a functional overlap of *CYP96B22* in the execution of penetration resistance during basal and nonhost resistance of barley against different *Magnaporthe* species.

## Background

*Magnaporthe oryzae* is a fungal plant pathogen which causes devastating diseases on several grass species including the cereals rice, wheat and barley [1]. On rice the disease is known as “rice blast” and it causes the most important economic losses in rice cultivation world-wide. Recently the fungus attracted increased attention because it has become a major problem in barley and wheat cultivation, especially in South America, where the disease is named wheat or head blast [2,3]. Thus, there is an urgent need for novel resistance traits which could be engineered into modern elite barley and wheat cultivars. As a potential source for these traits, we investigated the nonhost response of barley against non-adapted *Magnaporthe* isolates. This fairly understood phenomenon protects all barley cultivars from infection with *Magnaporthe* species that are pathogenic on other grasses like *Digitaria* or *Pennisetum*[4]. Generally, nonhost resistance is regarded as durable in the wild and effective against all genetic variants of a pathogen species [5].

Infection of barley with a *Magnaporthe* host or nonhost isolate starts when a three-celled conidium attaches to the barley leaf surface facilitated by formation of spore tip mucilage (for review see [6-8]). Adhesion of the subsequently formed germ tube is also accompanied by secretion of an extracellular matrix. At the tip of the germ tube a specialized cell, called an appressorium, is formed in which a high turgor is generated that supports mechanical penetration of the underlying epidermal tissue. The next infection stages are different between *Magnaporthe* host and nonhost isolates. The host pathogen invades epidermal cells with bulbous hyphae initiating a short biotrophic phase of colonization. This is followed by a switch to necrotrophy in the course of which the pathogen feeds on actively killed host cells [9]. The disease cycle is completed after sporulation takes place in lesions that become visible on infected leaves. By contrast, penetration of barley leaves by nonhost isolates is generally counterattacked by active defense reactions that block completion of the pathogen’s life-cycle [4]. These reactions may act either during or after penetration and therefore contribute to the penetration and post-penetration defense repertoire, respectively. For barley, a typical plant reaction associated with penetration resistance against different *Magnaporthe* species is the accumulation of autofluorescent material at the side of attempted penetration beneath a fungal appressorium which is associated with the formation of a cell wall apposition, a so-called papilla [4,10]. Diminishing the effectiveness of penetration resistance, e.g. by interfering with the actin cytoskeleton or by using barley mutants, revealed that a hypersensitive reaction of attacked epidermal cells functions as a second line of defense, similar to a backup strategy [11]. The kinetic of the latter post-penetration resistance response suggests that it acts before the fungus switches its life-style to necrotrophy.

While much is known about signal exchange between hosts and pathogens during and after penetration, it is still uncertain whether signaling occurs beforehand at the surface. For barley the accumulation of novel mRNAs was shown at 4 hours after inoculation with the wheat powdery mildew fungus [12]. At this stage of infection the barley nonhost pathogen had not yet formed appressorial germ tubes, however penetration attempts by the primary germ tube could not be excluded. For rice it was reported that a transcriptional up-regulation of genes takes place at 16 hours after inoculation with *M. oryzae*, a timeframe at which the fungus had formed germ tubes and appressoria [1]. A potential source for signal components which may be released during the early stages of infection is the cuticle which represents the layer of the leaf surface firstly touched by pathogens [13]. The cuticle mainly consists of cutin, a polymer built from C16 and C18 fatty acid units. Intra-cuticular and epi-cuticular waxes are embedded in the cutin polymer matrix or deposited on its outer surface, respectively [14]. Generally, cuticular waxes are a complex mixture of long-chain aliphatic lipids with chain lengths from 26 to 34 C-atoms, triterpenoids and other cyclic compounds, all of which could be released by solvent extraction [14,15]. The very long chain fatty acids (VLCF, C26 to C34) are elongated in the epidermal ER starting with C16 and C18 fatty acids. This elongation is catalyzed by an elongation enzyme-complex consisting of four different enzymes [13]. Thereafter the VLFCs are either processed to primary alcohols and wax-esters in the acyl reduction pathway or reduced to alkanes in the decarbonylation pathway ( [13], see Additional file 1: Figure S1). Alkanes might then be further oxidized to secondary alcohols and ketones by the activity of the enzyme mid-chain alkane hydroxylase (MAH). In Arabidopsis a cytochrome P450 protein of the CYP96 gene family, designated CYP96A15, was found to have MAH-enzymatic activity [16]. Interestingly, inflorescence stems of all Arabidopsis *mah1* mutant lines were covered with a visually normal wax surface although they were devoid of secondary alcohols and ketones in comparison to the wild-type suggesting a high grade of plasticity in the formation of wax layers.

Here, we report on a barley cytochrome P450 gene which shows greatest homology to CYP96 family members. The gene was identified in a large transcriptome analysis to be specifically up-regulated after inoculation of barley plants with a *Magnaporthe* nonhost isolate [17]. Our results indicate that the gene was expressed in response to the ectoparasitic growth of the pathogen on the leaf surface and functional gene silencing analysis suggests its involvement in execution of penetration resistance.

## Results

### Infection of barley with a *Magnaporthe* nonhost isolate induces expression of *CYP96B22*

**Figure 1:**
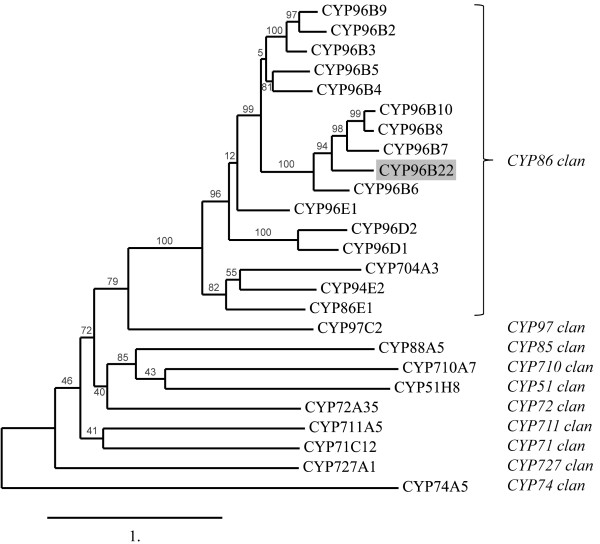
**Phylogenetic tree of selected rice CYP sequences and CYP96B22.** Protein sequences from rice CYP members were obtained from the Cytochrome P450 homepage [19] and used as a query for a homology search of the barley CYP sequence designated CYP96B22. The phylogenetic analysis was performed on the Phylogeny.fr platform [58] using the maximum likelihood method implemented in PhyML. The scale bar indicates 1.0 substitution per site, branch support values are given in percent. To simplify the tree view only members of the rice CYP96 family and selected representatives of the other families in the CYP86 clan and additional CYP clans are shown.

In a previous macroarray-based study, we had identified the barley IPK CR-EST clone HO07G08 to be among the three genes with the highest differential transcript abundance in the barley-*Magnaporthe* nonhost interaction as compared to host interaction [17]. A BLASTN search against the high confidence sequences of the recently published barley genome identified HO07G08 as part of the MLOC_15925.1 cDNA ( [18], see Additional file 1: Figure S2). The amino acid sequence deduced from HO07G08 is almost identical to the C-Terminus of the MLOC_15925.1 protein. Differences led to three single amino acid changes in the deduced proteins which might be due to differences among cultivars used. The MLOC_15925.1 protein is annotated as a cytochrome P450 (CYP) family protein. A search for conserved domains within the entire 61.3 kDa protein deduced from MLOC_15925.1 revealed a transmembrane region and a mid-chain hydroxylase CYP domain. A phylogenetic analysis of the protein with rice CYP sequences deposited at the Cytochrome P450 homepage [19] grouped the protein within the CYP96 family of the CYP86 clan (Figure 1). The sequence shares 67% identity to CYP96B13 of *Brachypodium distachyon* and 59% identity to CYP96B6 of rice (pers. com. D. Nelson). As suggested in Bak *et al.*[20], the sequence was sent to David Nelson, CYP nomenclature committee, who named it CYP96B22.

### Ectoparasitic growth of *Magnaporthe* on barley induces expression of *CYP96B22*

**Figure 2:**
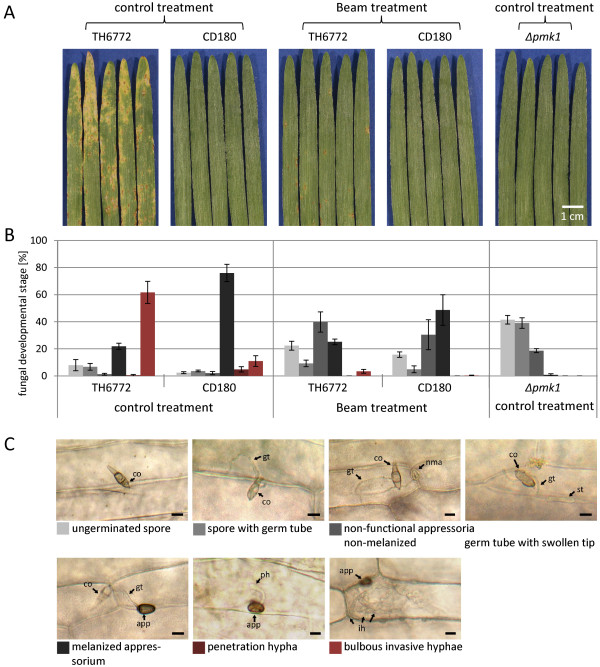
**Macroscopic and microscopic phenotypes of barley inoculated with *Magnaporthe *host (TH6772) or nonhost (CD180) isolates. A)** Macroscopic disease symptoms on primary leaves of barley cv. Vada at five days after inoculation with isolate TH6772, CD180 or mutant *∆pmk1* as indicated. Some of the plants were treated with the fungicide Beam prior to inoculation with isolate TH6772 or CD180. **B)** In the same experiment primary leaves of treated plants were harvested at 96 h p.i. for microscopic analysis. Interaction sites on plants were grouped into six categories according to the development of fungal infection structures as depicted in **(C)**. The *∆pmk1* mutant showed swollen structures at the tip of its germ tubes which were classified, similar to the non-melanized appressoria after Beam-treatment, as non-functional appressoria. From at least three leaves per treatment 100 interaction sites were inspected. The figure shows the mean frequencies for each category and the standard error from one representative experiment. The results were confirmed in two further biological replicates. scale bar: 10 μm; co: conidium; gt: germ tube; nma: non-melanized appressorium; st: swollen structure at the end of germ tube; app: appressorium; ph: penetration hypha; ih: invasive hyphae.

**Figure 3:**
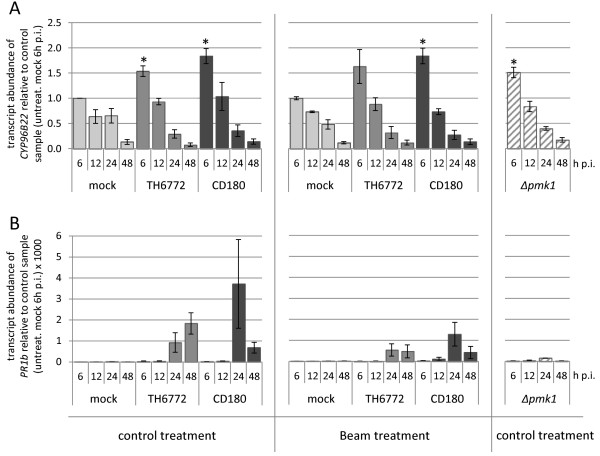
**Transcript accumulation in barley inoculated with different *Magnaporthe *isolates and pre-treated with the fungicide Beam.** Using RT-qPCR the relative transcript abundance of *CYP96B22***(A)** and *PR1b***(B)** in barley epidermal strips at 6, 12, 24 and 48 h after inoculation with a *Magnaporthe* host (TH6772) or nonhost isolate (CD180) or a mutant (*∆pmk1*) was determined. One group of plants was treated with the fungicide Beam prior to inoculation and the other with water (control treatment). Transcript abundance was calculated for each sample relative to the reference gene *EF1α* and is shown as the mean and standard error of three independent biological replicates. For normalization of differences in the absolute values among the biological replicates, the expression values were calculated in each experiment relative to the mock-inoculated sample (control treatment) at 6 h p.i. which was set to 1.0. Statistical analysis of *CYP96B22* expression was done only for the 6 h p.i. time point by comparing values to the mock-inoculated sample of the control treatment at 6 h p.i. using a paired t-test. Samples are marked with an asterisk in case of significant differences at P < 0.05.

At first, we confirmed the results obtained in the macroarray study on the differential expression pattern of *CYP96B22* in response to infection with different *Magnaporthe* isolates in independent experiments. Therefore, barley cultivar Vada was inoculated with the *M. oryzae* host isolate (TH6772) which resulted in clearly visible disease symptoms on primary leaves at five days after inoculation (Figure 2A). At the same time, leaves were inoculated with a *Pennisetum*-infecting nonhost isolate (CD180) and, as expected, did not show any disease symptoms even after a prolonged incubation period (Figure 2A). According to Zellerhoff *et al.*[4] and Faivre-Rampant *et al.*[1] both isolates are most likely members of different *Magnaporthe* species. For RT-qPCR analysis the epidermis from infected leaves of the same experiments was peeled (Figure 3). *CYP96B22* expression in mock treated samples was higher at early time points, i.e. at 6 and 12 h p.i. and decreased thereafter (Figure 3A). Essentially transcript profiles of *CYP96B22* after inoculation with the host or nonhost isolate followed the same pattern. However at 6 h p.i. the magnitude of expression was significantly higher in the host as compared to the mock situation and even higher in the nonhost interaction (Figure 3A). Thereby our data perfectly resembled the previously published results on the differential expression of *CYP96B22*[17].

The observed early induction of *CYP96B22* expression after *Magnaporthe* inoculation raised the question of the stimulus which caused this response. To discern the cellular events taking place during the early barley-*Magnaporthe* interaction transgenic, fluorescent-labeled *Magnaporthe* isolates were generated for both the host and nonhost isolate and inoculated onto barley plants. Analysis using epifluorescence microscopy revealed that at 6 h p.i. the majority of conidiospores were in the stage of germination while only a few already had formed appressoria (Additional file 1: Figure S3). Penetration pegs or invasive hyphae inside attacked epidermal cells were not observed before 24 h p.i either in the host or the nonhost interaction. It was concluded, therefore, that under our experimental conditions penetration took place between 12 and 24 h p.i. This, in turn, led to the conclusion that *CYP96B22* transcript accumulation at 6 h p.i. was triggered during the ectoparasitic growth of *Magnaporthe* on the surface of barley leaves and possibly before penetration.

### *CYP96B22* expression caused by *M. oryzae* does not require penetration

To further test the hypothesis that *CYP96B22* expression in barley is triggered before penetration, we made use of a fungicidal treatment and a penetration deficient *M. oryzae* mutant which prevent pathogen invasion after or before appressorium formation, respectively.

The fungicide Beam has the active ingredient tricyclazole which inhibits the reduction of 1,3,8-trihydroxynaphtalene to vermelone in the melanin biosynthetic pathway [21]. The absence of melanin then leads to *Magnaporthe* appressoria unable to penetrate and Beam is therefore widely used in rice cultivation to control blast disease. Here, we applied Beam by soil drench to barley plants prior to inoculation with *Magnaporthe* host or nonhost isolates. Beam-treated plants showed significantly less disease symptoms after inoculation with the *M. oryzae* host isolate TH6772 in comparison to the respective control indicating the effectiveness of the fungicide (Figure 2A). Microscopic analysis revealed that both the host and nonhost isolate developed infection structures on the leaf surface of Beam-treated plants up to the stage of appressorium formation (Figure 2B,C). Thereafter, as anticipated, Beam-treatment led to the absence of dark-pigmentation, i.e. melanization, in appressoria which was found for approximately half of all incidences. Almost no penetration pegs or invasive hyphae were found in epidermal cells of Beam-treated plants. It was concluded, therefore, that even in those cases in which dark pigmentation of appressoria was observed the fungus was mostly unable to penetrate (Figure 2B). The observation of few cells in which invasive hyphae were detected in the host interaction is consistent with the rare disease symptoms observed on the Beam-treated plants (Figure 2A) and indicated that the fungicide concentration used in our experiments did not mediate a 100% protection. Despite the blocked penetration on Beam-treated plants, *CYP96B22* transcripts did still accumulate at 6 h p.i. in response to inoculation with the host or nonhost isolate and in comparison to the respective control (Figure 3A). To further confirm this observation, we made use of the *M. oryzae* mutant *Δpmk1* which lacks a functional conserved mitogen-activated protein kinase [22]. This mutant cannot form normal appressoria and is therefore unable to penetrate rice or barley leaves. Since the mutant was generated in the genetic background of *M. oryzae* isolate Guy11, we firstly verified that Guy11 resembled the infection phenotype of isolate TH6772 on barley cv. Vada (data not shown). In agreement with Xu and Hamer [22], *Δpmk1* was found to be apathogenic on barley (Figure 2A). Similarly, microscopic evaluation confirmed the results of these authors by showing that the mutant only formed melanin-less, swollen structures instead of mature appressoria at the tip of their germ tubes (Figure 2C). Barley epidermis inoculated with *Δpmk1* was harvested in the same time-course experiments as for the Beam approach and RT-qPCR experiments showed enhanced expression of *CYP96B22* at 6 h p.i. (Figure 3A). Taken together our experiments with Beam and the *Δpmk1* mutant undoubtedly revealed that *Magnaporthe* triggers *CYP96B22* expression in barley independent from appressorium formation or successful penetration in both the host and nonhost interaction.

For comparison the expression of *PR1b* was monitored in the same set of RT-qPCR samples. In accordance with results published by Jarosch *et al.*[23], *PR1b* transcripts strongly accumulated in barley leaves in response to inoculation with the *Magnaporthe* host isolate (Figure 3B). By contrast, this strong *PR1b* transcript accumulation was neither seen in Beam-treated plants nor in response to the inoculation with the *Δpmk1* mutant (Figure 3B), indicating that induction of *PR1b* expression depends on the formation of mature appressoria and/or successful penetration.

### Knock-down of *CYP96B22* expression led to compromised penetration resistance

**Figure 4:**
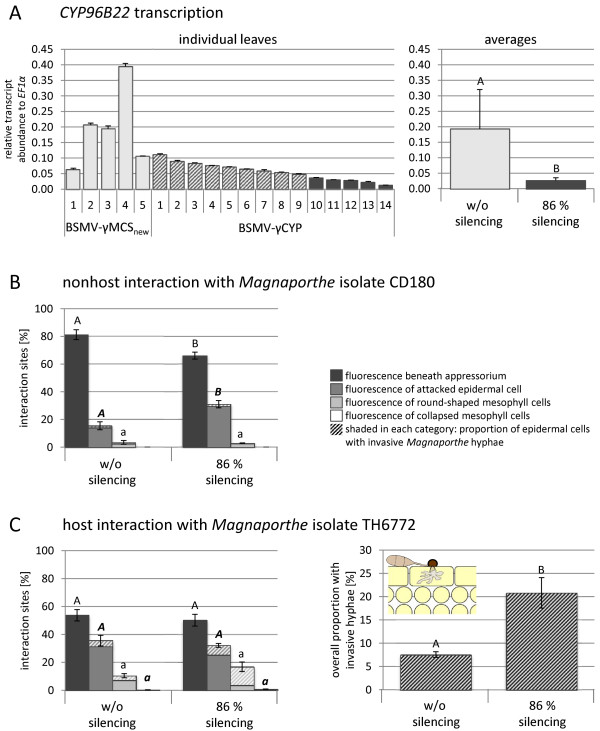
**BSMV-mediated silencing of *CYP96B22 *during infection of barley with *Magnaporthe*.** Primary leaves of barley plants were infected with BSMV without (BSMV-γMCS_new_) or with a silencing construct against *CYP96B22* (BSMV-γCYP). Plants showing virus symptoms were inoculated after two weeks on their third leaf with *Magnaporthe* isolates CD180 or TH6772 in detached leaf assay. **A)** Transcript abundance of *CYP96B22* was monitored by RT-qPCR in plants at 24 h p.i. with isolate CD180. Values shown are mean and standard deviation of two technical replicates and were calculated relative to the reference gene *EF1α* (2^(Ct(*EF1α*) – Ct(*CYP96B22*))^). For microscopic analysis two groups of plants were built, one consisting of the five plants inoculated with BSMV-γMCS_new_ and the other encompassing the five plants showing the highest *CYP96B22* transcript reduction (in average 86%, significance according to paired t-test). Cytological evaluation was done in the nonhost **(B)** and host **(C)** interaction at 96 and 48 h p.i., respectively. Therefore each infection site was assigned to different categories of plant defense responses which are associated with the deposition of autofluorescent material (for details see [4]). For both groups of plants 100 interaction sites were evaluated on each of the five leaves (means and standard errors are shown). Each category of plant defense responses was tested for significant differences between plants with and without silencing using t-test (P < 0.05). Significant differences were marked by different letters. In addition each attacked epidermal cell was investigated for the presence of invasive hyphae, frequencies are displayed by shading of bars. For clarity, the frequency of epidermal cells with invasive hyphae was depicted seperately for the host interaction. For the nonhost interaction **(B)** results were reproduced in two independent experiments. Higher invasive growth of the host isolate in silenced plants was confirmed in one biological replicate **(C)**.

Our next aim was to functionally evaluate whether *CYP96B22* has a crucial role in the defense of barley against different *Magnaporthe* isolates. For this purpose, we used a transient *Barley stripe mosaic virus* (BSMV)-induced gene silencing assay which was first described by Holzberg *et al.*[24] and which we had already adapted for *Magnaporthe* infection assays [25]. We inserted a 234 bp fragment of the *CYP96B22* coding sequence into the γ-subunit of BSMV. Using the si-Fi software, this construct was predicted to lead to efficient degradation of the *CYP96B22* mRNA and not to target transcripts of other barley genes (so-called off-targets) (Additional file 1: Figure S4). Infection of barley plants with BSMV exhibiting the silencing construct (BSMV-γCYP) or without it (BSMV-γMCS_new_) was done on primary leaves. Typical, mosaic-like, viral disease symptoms arose on 10-30% of individuals of both groups at 10–14 days after inoculation with BSMV, indicative of a successful virus infection. The third leaves of plants showing virus symptoms were cut off and divided into three equally long pieces (for experimental design see Additional file 1: Figure S5). Two of them were inoculated with *Magnaporthe* isolate CD180 and one with TH6772 in a detached leaf assay. Since previous studies showed that the knock-down of gene expression in BSMV-VIGS experiments can vary between different plants, silencing efficiency was monitored by RT-qPCR using leaf material harvested 24 h after inoculation with the nonhost isolate (Figure 4A). On average, the expression level of *CYP96B22* was lower in plants inoculated with the BSMV-γCYP than in BSMV-γMCS_new_-infected control plants.

For microscopic evaluation plants were grouped (Figure 4A, right figure), thereby one group consisted of the five plants inoculated with BSMV without silencing construct (BSMV-γMCS_new_) and the other encompassed those five plants showing the lowest *CYP96B22* transcript abundance after inoculation with BSMV with silencing construct (BSMV-γCYP). In the latter group transcript abundance was reduced by 86% as compared to plants of the first group (Figure 4A, right figure). At least 100 infection sites were screened for each plant from both groups and assigned to categories reflecting typical plant defense responses (for details see Zellerhoff *et al.*[4]). Above that, the presence of invasive hyphae in an attacked epidermal cell was recorded for each infection site. In the nonhost interaction with isolate CD180, the vast majority of attacked epidermal cells in the group of plants without silencing showed a local accumulation of autofluorescent material beneath appressoria associated with the formation of a cell wall apposition called papilla (Figure 4B). Invasive hyphae were never observed in these cells indicating an efficient defense response. Silencing of *CYP96B22* led to a significant reduction of infection sites grouped in this category. Instead a significant higher frequency of attacked epidermal cells was found showing autofluorescence of the entire cell wall which was verified in two independent replicate experiments. According to Zellerhoff *et al.*[4] this interaction category could be interpreted as post-penetration plant defense response. This was concluded from a time course experiment where the authors showed that, after successful penetration of ineffective papillae, invasive hyphae of *Magnaporthe* nonhost isolates were ultimately arrested in epidermal cells showing autofluorescent cell walls. The frequency of infection sites grouped within the other two categories did not differ significantly between plants of both groups (Figure 4B).

In the host situation no significant differences were observed among frequencies for each category between plants with or without efficient *CYP96B22*-silencing (Figure 4C). However, monitoring the invasion success, *CYP96B22*-silencing led to a higher frequency of invasive hyphae at infection sites where the fungus had successfully penetrated papillae (Figure 4C, right figure). In a biological replicate, we observed for the host interaction that *CYP96B22*-silencing also affected the transition of fungal development from epidermal to mesophyll tissue. This was evidenced by microscopy of infection stages at which the fungus had already built invasive hyphae in more than 35% of attacked epidermal cells and which was correlated with significant more collapsed mesophyll tissue in plants with efficient *CYP96B22*-silencing in comparison to control plants (data not shown). The collapse of mesophyll tissue is an indication for advanced *M. oryzae* colonization [4].

**Figure 5:**
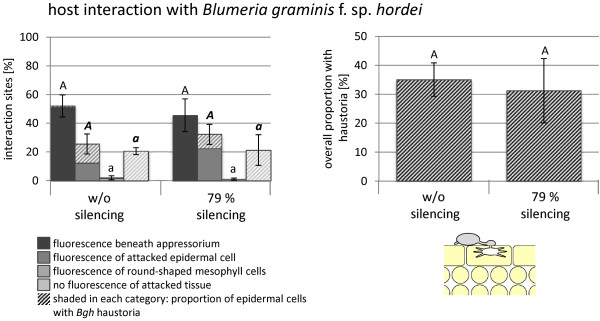
**Effect of *CYP96B22*-silencing in the barley-*Bgh *interaction.** Inoculation of plants with BSMV and grouping into two pools of plants with different expression level of *CYP96B22* was done as described for Figure 4. In this case the average silencing efficiency was 79%. Plants of both pools were inoculated with *Blumeria graminis* f. sp. *hordei* (*Bgh*) in a detached leaf assay. Infection sites were categorized according to defense responses associated with the deposition of autofluorescent material. For each leaf 100 infection sites were evaluated at 48 h after inoculation and the mean and standard error of four leaves are presented. Attacked epidermal cells were further investigated for the presence of mature haustoria (see schematic drawing in the lower right corner) and the frequencies are displayed by shading of bars. Cells found with haustoria were summed up as shown in a separate diagram in the upper right corner. Each category of plant defense responses was tested for significant differences between plants with and without silencing using t-test (P < 0.05) and this revealed no significant differences. It was confirmed in an independent experiment that *CYP96B22* silencing had no effect on the frequency of haustoria.

To investigate whether the effect of *CYP96B22*-silencing on enhanced penetration success also applies for other pathogens, VIGS experiments were conducted with the barley powdery mildew fungus *Blumeria graminis* f. sp. *hordei (Bgh)*. In this case, the group of plants chosen for microscopic evaluation showed 79% less transcript abundance for *CYP96B22* in comparison to the group of plants inoculated with BSMV without silencing construct (Figure 5). However, substantial differences were observed neither among the different categories of plant defense reactions nor in the frequency of successfully formed haustoria.

## Discussion

During recent years, large transcriptome profiling has become a routine analysis in the study of plant responses against abiotic and biotic stresses and was considered as a promising tool on the way to improve crop performance [26]. Here, we report on a cytochrome P450 gene of barley which was identified in a macroarray-based study as differentially regulated during nonhost and host interactions with fungal isolates of the *Magnaporthe* species complex. We provide evidence that this gene is involved in execution of penetration resistance as an early response to the ectoparasitic growth of the pathogen on the leaf surface.

Starting in 2000, the Leibniz Institute of Plant Genetics and Crop Plant Research (IPK) at Gatersleben developed a macroarray on the basis of expressed sequence tags (ESTs) and made it available as a resource for the barley community to study gene expression in response to various treatments [27]. Using this array, we identified a set of barley genes whose transcripts accumulate to a higher dose or at earlier time points after inoculation with a *Magnaporthe* nonhost as compared to a *Magnaporthe* host isolate [17]. A prominent member among these genes was an EST clone identical to the barley MLOC_15925 cDNA which has a domain characteristic for cytochrome P450 enzymes (Additional file 1: Figure S2). Plant P450s are involved in primary and secondary metabolic processes and catalyze different monooxygenation and hydroxylation reactions [28]. The wide distribution of P450s in a plant species-specific manner implies that they are subject to high evolutionary pressure which led to diversification and finally to novel metabolic pathways and products with acquired new biological functions. Search for homology among plant P450s placed the MLOC_15925 protein into the CYP96 family (designated therefore *CYP96B22*, Figure 1) and suggested an enzymatic function as midchain alkane hydroxylase (MAH). The closest Arabidopsis homologue of CYP96B22 is CYP96A10 (At4g39490), which was shown to be a splicing variant of a monocistronic transcript, forming a dimer with CYP96A9 [29,30]. It was suggested that the dimerization is required for the modification of hydrophobic substrates [29]. In Arabidopsis *CYP96A10* transcripts were found less abundant than *CYP96A9* transcripts [30], accounting for *CYP96A10* concentration as a rate limiting factor at least at the level of transcriptional regulation. Assuming a similar situation for barley, this could be interpreted as if *CYP96B22* has gained a novel regulatory function in the resistance response of barley against *Magnaporthe* nonhost isolates. In this scenario, the transcriptional up-regulation of *CYP96B22* will lead to enhanced formation of heterodimers with a putative barley protein homologous to CYP96A9 and finally to more production of a hydrophobic product.

So far the only CYP96 enzyme characterized in more detail is Arabidopsis MAH1 which provides secondary alcohols and ketones as building blocks for the cuticular wax layer (Additional file 1: Figure S1; [16,31]). This outermost surface layer is the first barrier which plant pathogens have to cope with during infection [13]. Additionally, the cuticle might also be a reservoir for the release of signal molecules which could be perceived by the attacked plant itself as damage-associated molecular patterns (DAMP, [32]) leading to the execution of defense responses. Alternatively, cuticle constituents could be used by pathogens as host identification factors which was shown e.g. in the barley-powdery mildew interaction for a C26-aldehyde [33]. Cuticle components may also function in bacterial pathogenicity, e.g. it was shown that the Arabidopsis *CYP86A2,* a plant P450 which is involved in cuticle development, is required for the induction of the bacterial type III secretion system [34]. On the other hand it was shown that free cutin monomers, most likely released by the activity of fungal derived cutinases, may act as endogenous DAMPs and induce plant defense responses [35]. In Arabidopsis the main epicuticular wax components are alkanes, secondary alcohols and ketones with a chain length of C29, all of which are synthesized via a MAH1-dependent pathway [36]. However, barley cuticles mainly contain a C26-primary alcohol, n-hexacosanol, whose synthesis does not require a MAH1 enzymatic conversion [33]. It might be, therefore, that MAH1-derived wax components in barley are less important against pathogen attack as structural barriers but instead act in signaling.

We addressed the biological function of *CYP96B22* in a transient assay using BSMV as a tool to knock-down gene expression (Figure 4). The usefulness of BSMV-mediated VIGS for functional genomics in wheat and barley has been demonstrated in several recent publications [37,38] and we already adopted the assay for the study of barley-*Magnaporthe* interactions [25]. Since it was shown for wheat that BSMV-infection prior to inoculation with *M. oryzae* host isolates may lead to decreased penetration [39], we used in all of our VIGS-experiments plants inoculated with BSMV without silencing construct as an internal control (Figure 4). Doing so and comparing these control plants with those showing the highest reduction in *CYP96B22* transcript abundance, a significant decrease in the frequency of papillae at sites of attempted penetration by the *Magnaporthe* nonhost isolate CD180 was observed (Figure 4). Since this latter result went along with more epidermal cells showing deposition of autofluorescent material at entire cell walls, this is indicative of ineffective papillae which could not block *Magnaporthe* penetration [11,23,40]. The more rapid transition of fungal invasion across cell walls became even more obvious in the barley host interaction with *M. oryzae* isolate TH6772 where *CYP96B22* silencing led to an increase of invasive hyphae in attacked epidermal cells. Similar findings for decreased penetration defense and more frequent entry of pathogens into epidermal cells, were also reported for Arabidopsis *pen*-mutants (for review see [41]). In the light of this finding *CYP96B22* could be regarded as being a barley *PEN*-gene. However, neither in Arabidopsis *pen*-mutants nor in our experiments with *CYP96B22*-VIGS, nonhost pathogens were able to sporulate, indicating effective post-penetration resistance mechanisms. Disabling this second line of defense by blocking the EDS1-PAD4-SAG101 signaling complex renders Arabidopsis fully susceptible even to non-adapted pathogens [42]. In barley, the deposition of autofluorescent material seems to be a cytological marker for this back-up strategy in interactions with *Magnaporthe*.

Nonhost resistance is generally considered to be determined by several quantitative trait loci all of which must act in common to prevent infection by a would-be pathogen [43]. Only the detailed monitoring of minor cytological changes in BSMV-VIGS treated barley plants enabled us to assign a function in penetration resistance against *Magnaporthe* nonhost isolates to the barley gene *CYP96B22*. It would be advisable, therefore, to refrain from macroscopic evaluation in nonhost analysis after knock-out or knock-down of candidate gene expression. Since the infection process of the adapted powdery mildew fungus on barley plants after knock-down of *CYP96B22* expression was not altered (Figure 5), we could rule out the possibility of an entire collapse of penetration resistance in these plants. The finding that *CYP96B22* may act differently in barley powdery mildew vs. *Magnaporthe* interactions was not astonishing, since similar observations had been made before for barley *MLO* or *ROP* genes [10,44]. This ambivalence in gene regulation was interpreted as part of a dedicated response of barley to biotrophic or hemi-biotrophic pathogens. Interestingly, *CYP96B22* was found to be down-regulated in barley after inoculation with *Puccinia triticina* and *Puccinia hordei*[17]. However a functional verification of this result e.g. in a BSMV-VIGS assay has not been reported yet.

An unexpected finding of the present study was the sensing of the ectoparasitic growth of *Magnaporthe* germ tubes at the leaf surface by barley plants. Using the *M. oryzae* mutant *Δpmk1*, we undoubtedly verified that penetration is not necessary to trigger *CYP96B22* expression (Figure 3). We showed also for *Magnaporthe* nonhost isolates, for which no *Δpmk1* mutants existed, that they were able to cause the same phenotype by using a fungicide leading to non-functional appressoria and thereby preventing penetration (Figures 2 and 3). Other studies also indicated a signal exchange between pathogens and plants before penetration, however the chemical nature of the signal itself was never elucidated [45-47]. Most recently it was shown for Arabidopsis that effectors of *Colletotrichum higginsianum* are secreted before penetration [48]. The homology of CYP96B22 to MAH1 enzymes and its implication in synthesis of cuticular waxes leads us to speculate that MAH1-dependent waxy components might be deliberated during the mucilage-facilitated attachment of spores and germ tubes to the leaf surface. Thereafter they are sensed as DAMPs and lead to increased defense responses associated with penetration resistance as e.g. the formation of effective papilla. In the host situation, *M. oryzae* isolates might then secrete effector molecules which negate these defense reactions [49].

## Conclusions

Our results provide functional evidence that the barley cytochrome P450 gene *CYP96B22* plays a crucial role in execution of the nonhost response of barley against pathogens from the *Magnaporthe* species complex. In the light of head blast as becoming a more prevalent disease in barley and wheat cultivation, our study might provide useful information for further breeding strategies. However, more studies are needed to establish whether the deduced protein functions in strengthening of structural barriers against pathogen attack or in generation of signal molecules.

## Methods

### Biological material and inoculation

*Hordeum vulgare* cultivars Ingrid, Vada and Morex were kindly provided by Paul Schulze Lefert (Max-Planck Institute for Plant Breeding Research, Cologne, Germany), Rients Niks (Wageningen University, Netherlands) and Patrick Schweizer (IPK Gatersleben, Germany), respectively. After 24 hours pre-germination on wet filter paper, germlings were transferred to standard soil (type ED73, Balster Einheitserdewerk GmbH, Froendenberg, Germany). For analysis of epidermal transcripts, 7×7×8 cm^3^ plastic pots with 10 germlings each were kept in a growth chamber with 16 h light- (200–250 μmol s^-1^ m^-2^) and 8 h dark-rhythm at 18°C and 65% relative humidity. For VIGS experiments, 10×10×10 cm^3^ pots with 4 germlings each were kept in a growth cabinet with a 16 h light period at 26°C and an 8 h dark period at 23°C.

The *Magnaporthe* isolates TH6772, CD180 and *∆pmk1* were kindly provided by the institute of Biochemistry, Tamagawa University (Machida-shi, Tokyo, Japan), D. Tharreau (CIRAD Montpellier, France) and N. J. Talbot (University of Exeter, UK), respectively. Transgenic *Magnaporthe* isolates containing either the DsRed (TH6772) or GFP (CD180) fluorescent protein were generated by *Agrobacterium*-mediated transformation using standard protocols as described in Rho *et al.*[50] and Tucker *et al.*[51]. Fungal cultures were maintained on oatmeal agar (20 g l^-1^ agar, 2 g l^-1^ yeast extract, 10 g l^-1^ starch, 30 g l^-1^ oatflakes) at 23°C in the dark. Sporulation was induced by cultivation of fungal cultures at a 16 h light/ 8 h dark regime under black light at 22°C. For sporulation isolate CD180 was transferred to new oatmeal agar plates and isolate TH6772 and the *∆pmk1* mutant were cultivated on rice leaf agar (water extract of 50 g rice leaves per litre, 2 g l^-1^ Faex medicinales [yeast extract; Biolabor, Bremen, Germany], 10 g l^-1^ water-soluble starch, 15 g l^-1^ agar). After 2 weeks conidia were harvested by rinsing the plates with water and filtering through three layers of gauze. For inoculation, the conidia were suspended in spraying solution (2 g l^-1^ gelatin, 1 ml l^-1^ Tween) at a concentration of 200,000 conidiospores ml^-1^ and sprayed onto barley leaves. After incubation at 24-26°C and 100% relative humidity for 24 h in the dark, inoculated plants were covered with a plastic hood and kept under growing conditions described above.

*Blumeria graminis* f. sp. *hordei* isolate K1 (obtained from Max-Planck-Institute for Plant Breeding Research, Cologne, Germany) was propagated on barley cv. Ingrid under growth conditions described above. Plants with powdery mildew pustules were shaken to remove older conidia one day prior to harvest of inoculum. For inoculation the freshly emerged conidia were then blown over leaf material in a settling tower. After 30 minutes the inoculation density was monitored with a standard Thoma counting chamber (approx. 10 spores mm^-2^) and the inoculated plant material was transferred to a growth cabinet.

### Sampling and RNA extraction for barley epidermal peels

Vada was inoculated seven days after sawing with mock solution or different *Magnaporthe* isolates. Three days prior to inoculation plants were drenched with 100 ppm Beam solution (received from BayerCropScience, Monheim, Germany) up to saturation of the soil volume and a control group of plants was treated with an equal amount of water. At 6, 12, 24 and 48 hours after inoculation (h p.i.) the abaxial epidermis of at least 20 primary leaves was peeled and directly frozen in liquid nitrogen. RNA was extracted using PeqGold RNApure (Peqlab, Erlangen, Germany).

### Determination of transcript abundance

For removal of genomic DNA, 1 μg RNA was treated with DNase I and reverse-transcribed to cDNA with primer HindAnchorT (Additional file 2: Table S1) using RevertAid Reverse Transcriptase. The cDNA of BSMV-infected plants was digested with RNase A and RNase H to remove viral RNA and afterwards cleaned up with Gene Jet Purification Kit (kits and enzymes were purchased from Thermo Fisher Scientific Bioscience GmbH, Schwerte, Germany). Reverse transcription quantitative PCR (RT-qPCR) was performed using SYBR Green qPCR SuperMix-UDG with ROX (Invitrogen, Carlsbad, USA) and gene-specific primers (Additional file 2: Table S1). *CYP96B22*-, *PR1b*- and *Ubiquitin*-specific primer sequences were designed with Primer3Plus [52]; primer sequences for *EF1α* and *GAPDH* were from McGrann *et al.*[53]. Specificity of all primers for the respective genes was confirmed by sequencing the resulting RT-qPCR products. The RT-qPCR was performed on an ABI Prism 7300 sequence detection system (Applied Biosystems, Life Technologies, Darmstadt, Germany). After the activation cycle at 50°C for 2 min. and at 95°C for 10 min., samples were exposed to 40 cycles of 15 s at 95°C and 1 min. at 60°C. Afterwards, a melt-curve analysis was performed for each sample. For analysis of relative transcript abundance only Ct-values with a target sequence-specific melting point were evaluated. According to Livak and Schmittgen [54] the transcript accumulation of the target gene was calculated relative to a reference gene (2^(Ct(reference) – Ct(target))^). For this study barley *EF1α* was chosen as reference gene because it showed the most stable expression in comparison to other tested reference genes (*Ubiquitin, GAPDH* and *EF1α*).

### Microscopic studies

For cytological analysis of plant-pathogen interactions, barley leaves were harvested and destained in 14% acetic acid in ethanol (v/v). For investigation of early fungal developmental stages, inoculated leaves were placed on Whatman paper soaked with 25% acetic acid in ethanol (v/v) until bleached. This method was first described by Carver and Ingerson-Morris [55] and prevents the removal of fungal infection structures during staining procedures. Bleached leaves were analyzed in water by brightfield and fluorescence microscopy (DMBRE, Leica Microsystems, Wetzlar, Germany). Structures of *Bgh* on the leaf surface were stained with 10% ink in 25% acetic acid (v/v) prior to microscopy. At least 100 individual infection sites were evaluated per leaf (Figure 2C) as described in Zellerhoff *et al.*[4]. Interaction sites in contact to stomata or vascular bundles were excluded from evaluation.

### Database analyses

The sequence of HO07G08 was taken from the IPK Crop EST database (http://pgrc.ipk-gatersleben.de/cr-est/) and used to BLASTN query the transcript sequences published by the International Barley Sequencing Consortium [18]. A conserved domain search was performed with InterProScan [56] and at NCBI [57]. Protein sequences of CYPs identified in rice were obtained from the Cytochrome P450 homepage [19]. A phylogenetic analysis of CYP96B22 and chosen rice sequences was performed on the Phylogeny.fr platform [58] and comprised an alignment of the sequences with MUSCLE (v3.7, default settings). The phylogenetic tree was reconstructed using the maximum likelihood method implemented in the PhyML program (v3.0 aLRT). The WAG substitution model was selected assuming an estimated proportion of invariant sites (of 0.015) and 4 gamma-distributed rate categories to account for rate heterogeneity across sites. The gamma shape parameter was estimated directly from the data (gamma = 2.977). Reliability for internal branch was assessed using the aLRT test (SH-Like). Graphical representation and edition of the phylogenetic tree were performed with TreeDyn (v198.3).

### *Barley stripe mosaic virus* – virus induced gene silencing (VIGS)

For VIGS studies DNA plasmids carrying full-length clones of BSMV isolate ND18 components (pT7-BSMV-α, pT7-BSMV-βΔCP, pT7-BSMV-γMCS and pT7-BSMV-γHvPDS400) were kindly provided by Merete Albrechtsen (University of Aarhus, Frederiksberg, Denmark). pT7-BSMV-γMCS was modified by insertion of a larger multiple cloning site (MCSnew, Additional file 2: Table S1) into *BamH*I restriction site. A 234 bp gene fragment of *CYP96B22* was amplified using the primer sequences given in Additional file 2: Table S1 and inserted in antisense orientation into *Eco*RI and *Apa*I restriction sites of pT7-BSMV-γMCSnew to form pT7-BSMV-γCYP. Target sequences of the VIGS construct were predicted with si-Fi software (version 3.1, http://labtools.ipk-gatersleben.de/). Plasmids were linearized and used as templates for *in vitro* transcription as described [59]. The viral RNA subunits (α, β and γ) were mixed equally to assemble infectious virus. After addition of four volumes of FES buffer (0.1 M glycine, 0.06 M K_2_HPO_4_, 1% NaPO_4_ (w/v), 2% Bentonite (w/v), 1% celite (w/v); pH 8.5-9.0), the virus was rub-inoculated onto five days old primary leaves of barley cv. Morex (25 μl were sufficient to inoculate 3–4 plants) which were subsequently washed with water. Two weeks after inoculation third leaves of plants showing virus symptoms were placed on kinetin agar (1 mg l^-1^) and inoculated with isolates of *Magnaporthe* or *Blumeria graminis* f. sp. *hordei,* respectively. Silencing efficiency was monitored for each plant by harvesting a part of inoculated leaves which was frozen in liquid nitrogen (see Additional file 1: Figure S5). RNA was isolated according to Voegele *et al.*[60]. Therefore, the material was ground in a mortar and suspended in 600 μl cell lysis buffer (20 g l^-1^ SDS ultrapure, 68 mM sodium citrate, 132 mM citric acid, 10 mM EDTA, pH 3.5). After precipitation of proteins and DNA by 5 min. incubation with 200 μl precipitation buffer (4 M sodium chloride, 17 mM sodium citrate, 33 mM citric acid, pH 3.5), RNA was precipitated from the supernatant with isopropanol, washed in 70% ethanol and solved in double-distilled water. Determination of transcript abundance was performed by RT-qPCR as described above.

## Abbreviations

BSMV: *Barley stripe mosaic virus*; VIGS: Virus induced gene silencing; CYP: Cytochrome P 450 enzyme; siRNA: Small interference RNA; GFP: Green fluorescent protein; DsRed: *Discosoma* red fluorescent protein; RT-qPCR: Reverse transcription quantitative PCR; Bgh: *Blumeria graminis* f. sp. *hordei*.

## Competing interests

The authors declare that they have no competing interests.

## Authors’ contribution

RD conducted most of the experimental work, participated in design of the study and drafted the manuscript. NZ established BSMV-VIGS and suggested to follow *CYP96B22* as interesting candidate gene for nonhost resistance. CF generated the BSMV-CYP silencing construct and was, as RS, involved in biological replicates of VIGS experiments. US conceived the study, participated in design and coordination of experiments, drafted and finalized the manuscript. All authors approved the final manuscript.

## Supplementary Material

Additional file 1: Figure S1 Simplified pathway of wax biosynthesis in Arabidopsis [61]. **Figure S2.** Multiple Alignment of MLOC_15925.1, HO07G08 and re-sequenced *CYP96B22* construct used in VIGS experiments. **Figure S3.** Time course study of the development of infection stages of *M. oryzae* on barley. **Figure S4.** Prediction of targets in the transcriptome of barley by the siRNA used in this study. **Figure S5.** Experimental design of VIGS experiments. Click here for file

Additional file 2: Table S1. Primer sequences used in this study. Click here for file
