# Supplementary material for: Ectoparasitic growth of Magnaporthe on barley triggers expression of the putative barley wax biosynthesis gene CYP96B22 which is involved in penetration resistance
Source: BMC Plant Biol. 2014 Jan 14;14:26. doi: 10.1186/1471-2229-14-26 (PMC3897914; doi:10.1186/1471-2229-14-26)
Supplement: Additional file 1: Figure S1 — Simplified pathway of wax biosynthesis in Arabidopsis [61]. Figure S2. Multiple Alignment of MLOC_15925.1, HO07G08 and re-sequenced CYP96B22 construct used in VIGS experiments. Figure S3. Time course study of the development of infection stages of M. oryzae on barley. Figure S4. Prediction of targets in the transcriptome of barley by the siRNA used in this study. Figure S5. Experimental design of VIGS experiments. [file 1471-2229-14-26-S1.pdf]

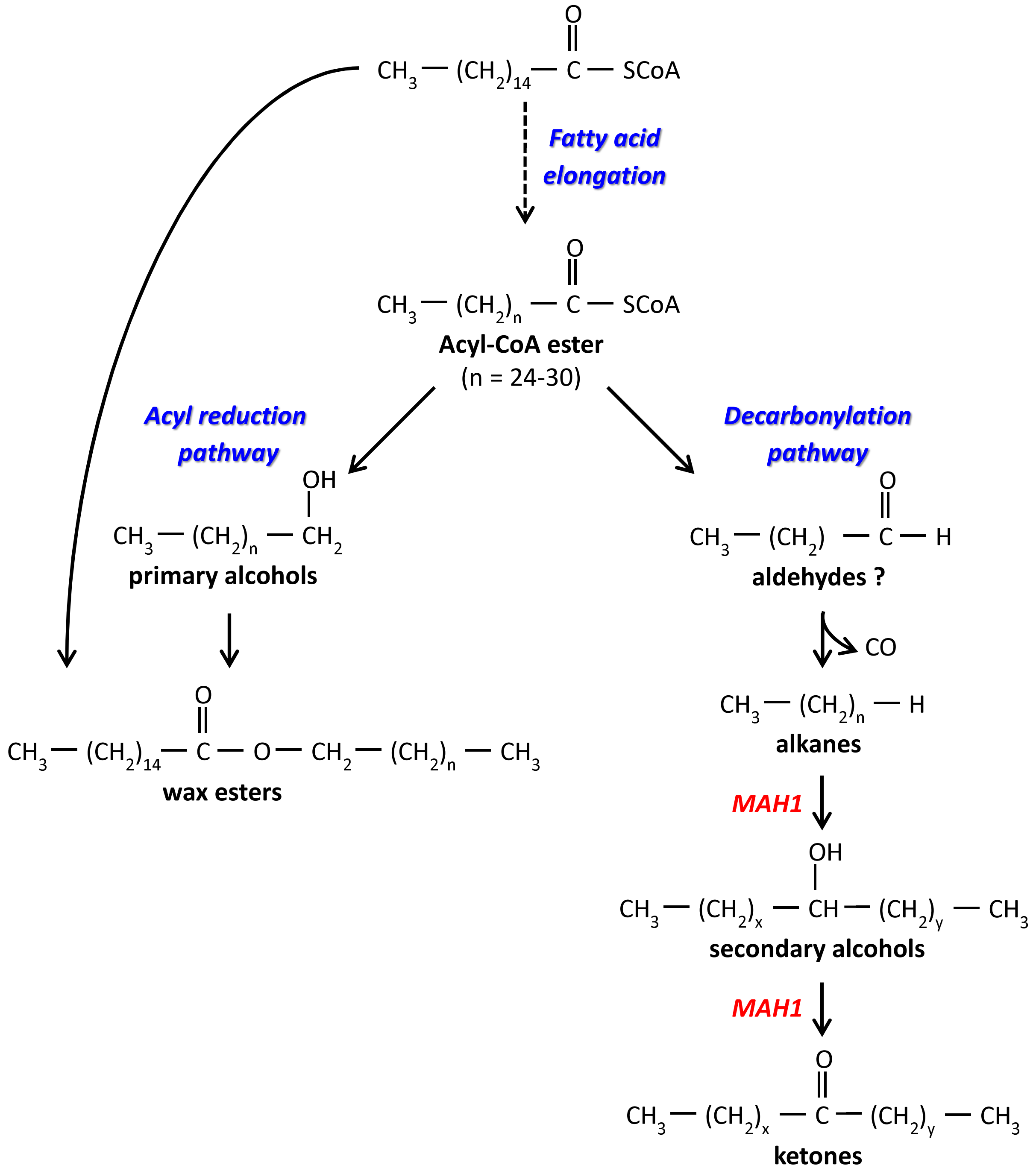

Figure S1: Simplified pathway of wax biosynthesis in Arabidopsis (redrawn from Greer *et al.* [16] and Samuels *et al.* [61]). The enzyme mid-chain alkane hydroxylase (MAH1) which, according to Greer *et al.* [16], catalyzes the enzymatic conversations of alkanes to secondary alcohols and ketones is highlighted in red.

```
MLOC_15925.1      1 atggctgctgcatgcatgccaccgagggaaacttctcaccatataaatggagccaaagccatgtccatgcagcataaacccaaagcacaa
HO07G08             ------------------------------------------------------------------------------------------
VIGS constr.        ------------------------------------------------------------------------------------------

MLOC_15925.1     91 gcatctccccatacactactcacaacaatgtctactatgtcattctcgcaagagctgctcatctccacgctcgccatcattcttgtttcc
HO07G08             ------------------------------------------------------------------------------------------
VIGS constr.        ------------------------------------------------------------------------------------------

MLOC_15925.1    181 atgtacacgttgtacctcaggtctagtagatcaaagggcccagcagtgctccccacaaactggccaatagtgggcgtgctcccctccctt
HO07G08             ------------------------------------------------------------------------------------------
VIGS constr.        ------------------------------------------------------------------------------------------

MLOC_15925.1    271 gtcgccaacctccacaacttgcacgactacctcgccgtcgtcctcgccggatcagggcacaacttcagggcaaccggcccgccggggacc
HO07G08             ------------------------------------------------------------------------------------------
VIGS constr.        ------------------------------------------------------------------------------------------

MLOC_15925.1    361 gggatgcggttcttcgtcacctgcgacccggccaacgtccggcacatcttcacgtccaaccacgcaaacttccccaagggcgcggagttc
HO07G08             ------------------------------------------------------------------------------------------
VIGS constr.        ------------------------------------------------------------------------------------------

MLOC_15925.1    451 gccgccatctttgacatcgcgggtggcagcttcttcaccactgaaggagagccctggcgtcggcagcgcacaagagcccagagagtcttg
HO07G08             ------------------------------------------------------------------------------------------
VIGS constr.        ------------------------------------------------------------------------------------------

MLOC_15925.1    541 agcaacccacggttggttgccactatgaccgcttgctgtcttgacaaggtggagaacggcctcctcccggtgctcatgcacatggcgacc
HO07G08             ------------------------------------------------------------------------------------------
VIGS constr.        ------------------------------------------------------------------------------------------

MLOC_15925.1    631 accgcgaccccgttcgacctgcaagctttgacaacgaggcttgtgttcgacataaccgccgcgccgctcttcggcgtcgacccgggcctc
HO07G08             ------------------------------------------------------------------------------------------
VIGS constr.        ------------------------------------------------------------------------------------------

MLOC_15925.1    721 ctgtcctcggacatgccgcccatggatgccgctgtcgccatggacacggtcatggaggtggccttgttccggcacatcgtgccggccact
HO07G08           1 ---------------------------------------------------------------------cggcac---------------
VIGS constr.        ------------------------------------------------------------------------------------------

MLOC_15925.1    811 ggctggaaggtgatgaggcggctaaacatcggcccggaaaggaagctcgccgcagcgcacaccgtgctgcgcgggttcgttgccgagatg
HO07G08           7 --------------gaggcggctaaacatcggcccggaaaggaagctcgcctcagcgcacacggtgctgcgcgggttcgttgccgagatg
VIGS constr.        ------------------------------------------------------------------------------------------

MLOC_15925.1    901 atggagaggaggaagatgaaggaacatgctgctgctggtgacgaggaagccccttcgtccgtggacattctgtcttcctacatcgacgat
HO07G08          83 atggagaggaggaagatgaaggaacatgctgctgctggtgacgaggaagccccttcgtccgtggacattctgtcttcctacatcgacgat
VIGS constr.        ------------------------------------------------------------------------------------------

MLOC_15925.1    991 ccagactaccagcagaacgatggcttgctccatgcgacgctcatcaactacatgatcgccgggagggacacgatcagcacagctttgaca
HO07G08         173 ccagactaccagcagaacgatggcttgctccatgcgacgctcatcaactacatgatcgccgggagggacacgatcagcacagctttgaca
VIGS constr.        ------------------------------------------------------------------------------------------

MLOC_15925.1   1081 tgggtcttctacaacctcgcccagaaccctcgggtcatgtcggtcatccgcaacgaactatcacccatcgcatcacggaaagcagccagc
HO07G08         263 tgggtcttctacaacctcgcccagaaccctcgtgtcatgtcggtcatccgcaacgaactatcacccatcgcatcacggaaagcagctagc
VIGS constr.      1 -------------------------------------------------------aactatcacccatcgcatcacggaaagcagctagc

MLOC_15925.1   1171 gatagcagcagcaccatggtgatctttgaacccgaggagaccaaatctcttgtctacatgagagctgccctacaggagtctctcaggttg
HO07G08         353 gatagcagcagcaccatggtgatctttgaaccggaggagaccaaatctcttgtcttcatgagagctgccctacaggagtctctcaggttg
VIGS constr.     36 gatagcagcagcaccatggtgatctttgaaccggaggagaccaaatctcttgtcttcatgagagctgccctacaggagtctctcaggttg

MLOC_15925.1   1261 cacccgccggtccccatcgagcgtaagacagcggtcgccagcgacgtgatgccgagtggccatgtggtgcatgccggtgacaccttattg
HO07G08         443 cacccgccggtccccatcgagcgtaagacagcggtcgccagcgacgtgatgccgagtggccatgtggtgcatgccggtgacaccttattg
VIGS constr.    126 cacccgccggtccccatcgagcgtaagacagcggtcgccagcgacgtgatgccgagtggccatgtggtgcatgccggtgacaccttattg

MLOC_15925.1   1351 atttctctccactccatggggagaatggaagacgtgtggggaaagactgccgggagtataacccagatagatggctctcagaggatggc
HO07G08         533 atttctctccactccatggggagaatggaagaggtgtggggaaagactgccgggagtataacccagatagatggctctcagaggatggc
VIGS constr.    216 atttctctccactccatgg-----------------------------------------------------------------------

MLOC_15925.1   1441 aagaagctgcggtacgtgccgtctcacaaattttggcattcagctccggcccgaggatttgccttggcaaggacatctcatttatgcag
HO07G08         623 aagaagctgcggtacgtgccgtctcacaaattttggcattcagctccggcccgaggatttgccttggcaaggacatctcatttatgcag
VIGS constr.        ------------------------------------------------------------------------------------------

MLOC_15925.1   1531 atgaatactattgtcgcggcaatggtgtggaacttcgatgtggagctggttgacggctcaaggtccagcccgagatgtcttgtgtactg
HO07G08         713 atgaatactattgtcgccgcaatggtgtggaacttcgatgtggagctggttgacggctcaaggtccagcccgagatgtcctgtgtactg
VIGS constr.        ------------------------------------------------------------------------------------------

MLOC_15925.1   1621 cgcatgaaaaatgggctcatggtgaagctgaagaagcgagaaatg---------------------------------------------
HO07G08         803 cacatgaaaaatgggctcatggtgaagctgaagaagcgagaaatgtgacctttaggcctccttttcttatttgtatcttgtgtgagcctt
VIGS constr.        ------------------------------------------------------------------------------------------

MLOC_15925.1        ------------------------------------------------------------------------------------------
HO07G08         893 tcggggttcgggacctcagggtatttgtatgtgtactgctataagtttttgtgtgcgaatgttttgctataataagttgataaatatacg
VIGS constr.        ------------------------------------------------------------------------------------------

MLOC_15925.1        ------------
HO07G08         983 tagaccctactt
VIGS constr.        ------------
```

Figure S2: Multiple Alignment of MLOC_15925.1, HO07G08 and re-sequenced *CYP96B22* construct used in VIGS experiments. The sequence of HO07G08 was taken from the IPK Crop EST database (http://pgrc.ipk-gatersleben.de/cr-est/) and used as BLASTN query against the transcript sequences published by the International Barley Sequencing Consortium [18]. The incomplete identity between MLOC_15925.1 and HO07G08 might be due to differences between barley cultivars or inaccuracies in the original sequencing of HO07G08 [27] or MLOC_15925.1. Identical parts of sequences are highlighted in yellow.

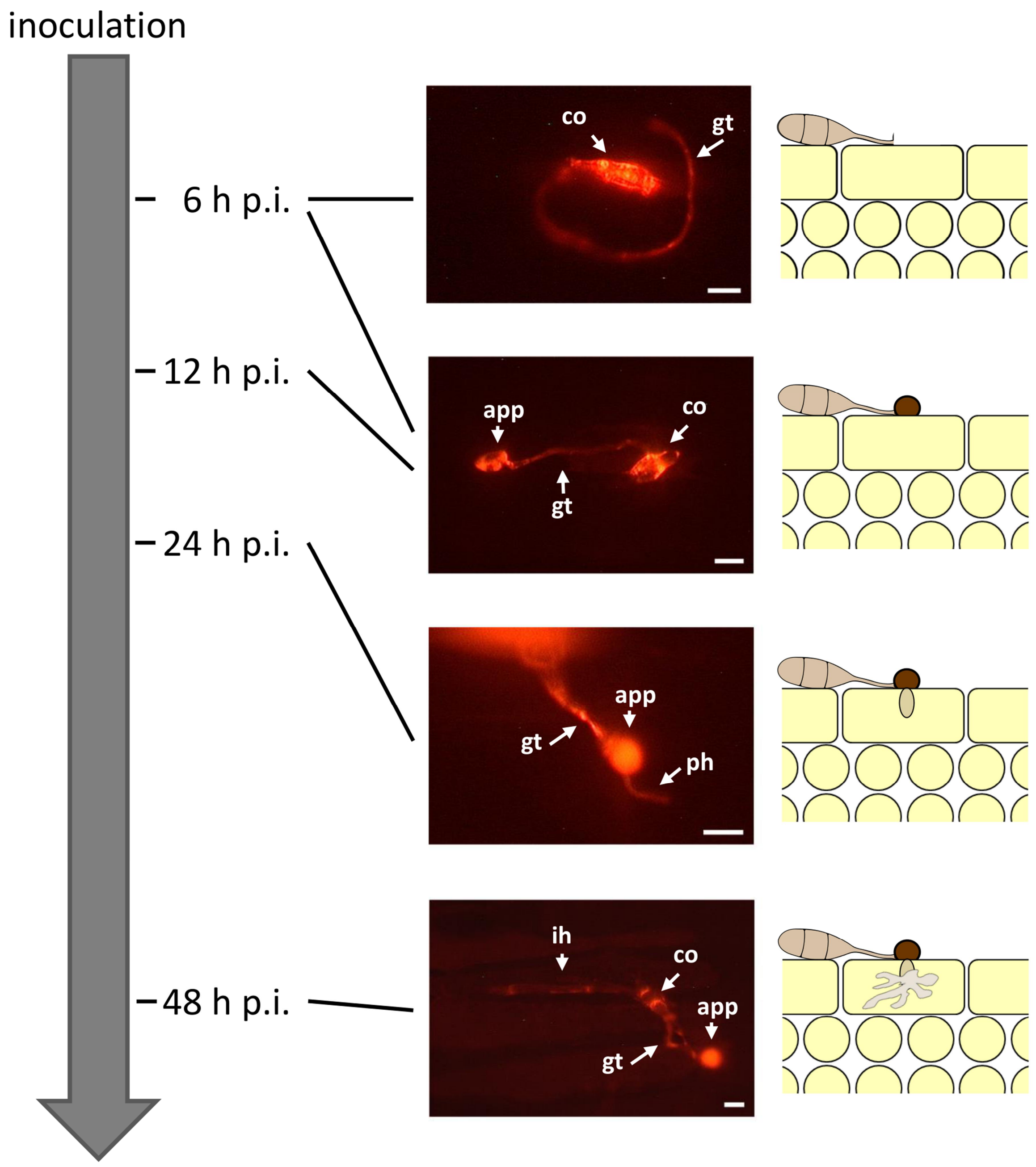

Figure S3: Time course study of the development of infection stages of *M. oryzae* on barley. Transgenic fungi labeled with DsRed were generated for the *M. oryzae* host isolate TH6772 and inoculated onto barley primary leaves. At 6 h after inoculation (h p.i.) germinated spores were found on the leaf surface some of which had already developed appressoria. At 12 h p.i. almost all germinated conidia had formed appressoria and at 24 h p.i. penetration hyphae got visible beneath appressoria which further developed into larger invasive hyphae detected around 48 h p.i. A similar kinetic was observed for the development of infection structures of a transgenic, GFP-expressing, nonhost *Magnaporthe* isolate (CD180), however in this case invasive hyphae were only rarely detected (data not shown). scale bar: 10 µm; co: conidium; gt: germ tube; app: appressorium; ph: penetration hypha; ih: bulbous invasive hypha

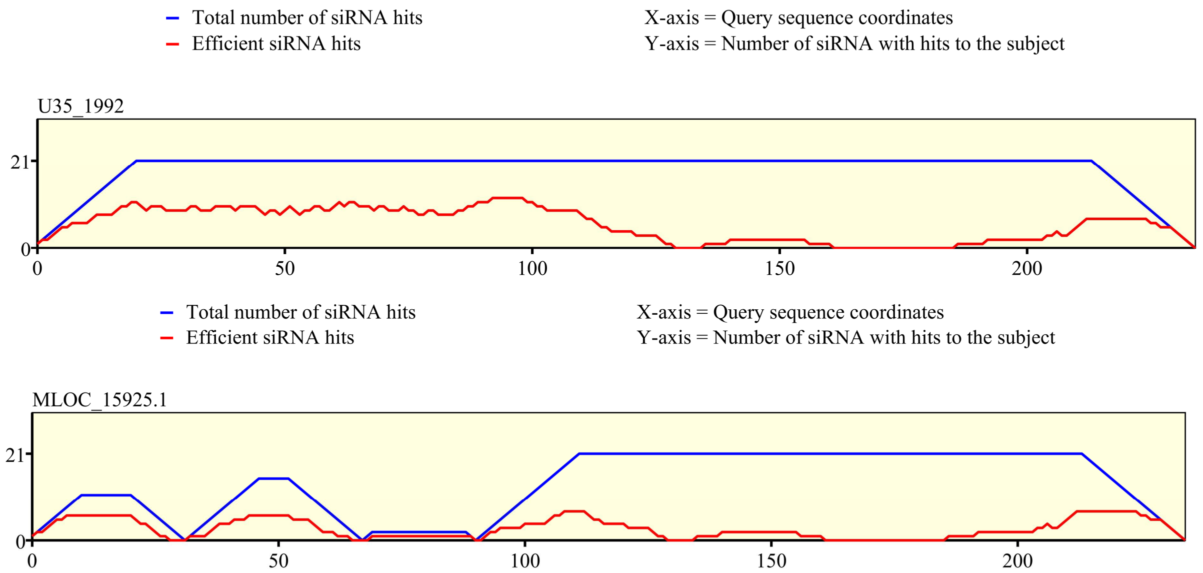

Figure S4: Prediction of targets in the transcriptome of barley by the siRNA used in this study. The target sequence for the *CYP96B22* silencing construct was predicted using the si-Fi software (http://labtools.ipk-gatersleben.de/). The 234 bp fragment of *CYP96B22* was used as a query to be scanned for potential generated siRNA and the predicted siRNA sequences were then checked for potential targets using the HarvEST database (assembly 35, http://harvest.ucr.edu/) or the high-confidence barley coding sequences [18]. In both databases a unique coding sequence was identified as potential target, both representing *CYP96B22* as deposited in HarvEST (U35_1992, http://harvest.ucr.edu/) or by Meyer *et al.* [18] (MLOC_15925.1).

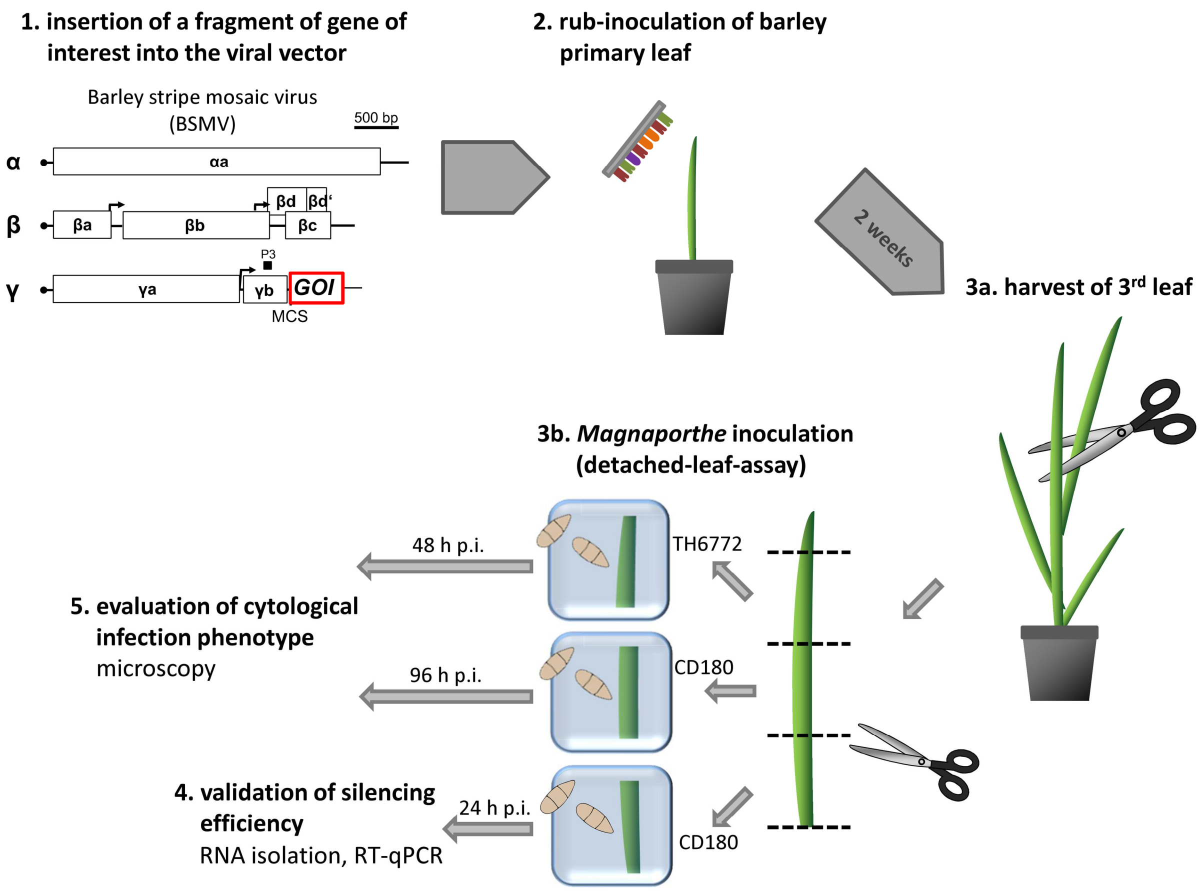

Figure S5: Experimental design of VIGS experiments.
A 234 bp fragment of *CYP96B22* was inserted into the γ-subunit of BSMV (1) (picture modified according to [59]). *In vitro* generated viral transcripts were rub-inoculated on barley primary leaves (2). Two weeks later the third leaf of plants showing viral disease symptoms was harvested and cut into three pieces, two of which were inoculated with the *Magnaporthe* nonhost isolate CD180 and one with the host isolate TH6772. Microscopic assessment of plant defense responses was done at time points indicated. Silencing efficiency was monitored by RT-qPCR using one leaf piece harvested at 24 h p.i. with CD180.
