# Supplementary material for: Ectoparasitic growth of Magnaporthe on barley triggers expression of the putative barley wax biosynthesis gene CYP96B22 which is involved in penetration resistance
Source: BMC Plant Biol. 2014 Jan 14;14:26. doi: 10.1186/1471-2229-14-26 (PMC3897914; doi:10.1186/1471-2229-14-26)
Supplement: Additional file 2: Table S1. — Primer sequences used in this study. [file 1471-2229-14-26-S2.pdf]

Table S1: Primer sequences used in this study

<table>
<tr><th colspan="2"></th><th>forward primer</th><th>reverse primer</th><th>product (bp)</th></tr>
<tr><td rowspan="6">for RT-qPCR</td><td>HindAnchorT</td><td>AAGCTTTTTTTTTTTTTTT(AGC)</td><td></td><td>cDNA of mRNA</td></tr>
<tr><td>PR1b</td><td>AACATCGTTGGACAGAAACCA</td><td>GGTGCATGAGATTAGGAACGA</td><td>98</td></tr>
<tr><td>CYP96B22</td><td>CTCCACATCGAAGTTCCACAC</td><td>CAAATTTTTGGCATTCAGCTC</td><td>109</td></tr>
<tr><td>Ubiquitin</td><td>TGAATGACCCAAGCATTCTCC</td><td>TCAATGTTGCTCATGGCTCTG</td><td>103</td></tr>
<tr><td>EF1α[a]</td><td>ATGATTCCCACCAAGCCCAT</td><td>ACACCAACAGCCACAGTTTGC</td><td>101</td></tr>
<tr><td>GAPDH[a]</td><td>CCTTCCGTGTTCCCACTGTTG</td><td>ATGCCCTTGAGGTTTCCCTC</td><td>124</td></tr>
<tr><td rowspan="2">for VIGS vector construction</td><td>MCSnew</td><td>CGCGGATCCGGGCCCCCGCCATGGCGGCCGCGGAATTCTCTAGAACTAGTGGATCCCGC</td><td>GCGGGATCCACTAGTTCTAGAGAATTCCGCGGCCGCCATGGCGGGGGCCCGGATCCGCG</td><td>59</td></tr>
<tr><td>CYP96B22</td><td>GCGGAATTCAACTATCACCCATCGCATCAC</td><td>TTATATGGGCCCCCATGGAGTGGAGAGAAATCA</td><td>234[b]</td></tr>
</table>

[a] primer sequences for *EF1α* and *GAPDH* are from McGrann et al. [53]

[b] product size without restriction sites
